# Photothermal fabrication of microscale patterned DNA hydrogels

**DOI:** 10.1098/rsos.171779

**Published:** 2018-02-28

**Authors:** Suguru Shimomura, Takahiro Nishimura, Yusuke Ogura, Jun Tanida

**Affiliations:** Graduate School of Information Science and Technology, Osaka University, Suita, Osaka, Japan

**Keywords:** optical fabrication, artificially engineered materials, photothermal effects, computer holography

## Abstract

This paper introduces a method for fabricating microscale DNA hydrogels using irradiation with patterned light. Optical fabrication allows for the flexible and tunable formation of DNA hydrogels without changing the environmental conditions. Our scheme is based on local heat generation via the photothermal effect, which is induced by light irradiation on a quenching species. We demonstrate experimentally that, depending on the power and irradiation time, light irradiation enables the creation of local microscale DNA hydrogels, while the shapes of the DNA hydrogels are controlled by the irradiation patterns.

## 1. Introduction

DNA hydrogels are artificial materials formed by linking together DNA motifs [1]. With suitably designed and modified DNA sequences, these hydrogels can exhibit desired properties and functions [2]. Gelation can be induced by changing the environmental conditions, including pH [3], temperature [4] or the insertion of specific molecules [5]. Designed sensing capabilities can be applied to a wide range of research fields, including tissue engineering and drug delivery. For example, DNA hydrogels can provide quantitative detection of thrombin [6]. Moreover, the control of their shape enables the manufacture of cell scaffolds to create cellular tissues [7]. By forming drops of two kinds of DNA solutions with small nozzles and then mixing them, DNA hydrogels can be formed.

In fabricating the specific morphology of DNA hydrogels, a mould with the corresponding shape is sometimes used [8]. However, to change the shape, a new mould is required. To accomplish the flexible design of DNA hydrogels, the patterning of two-dimensional structures on a substrate using a DNA initiator was reported by Wang *et al.* [9]. Furthermore, Li *et al.* [7] demonstrated the fabrication of three-dimensional structures using an ink-jet method. Although these methods can make complicated structures from DNA hydrogels, it takes a long time to form the desired shape, due to the necessity for coating DNAs on a glass slide or the slow scanning of the nozzles. A flexible and fast method to form DNA hydrogels is required.

Optical fabrication is a promising solution to this problem. Optical methods are widely used in material processing, because of their rapid processing times and ease of spatial control. The use of photomasks allows for the processing of complicated shapes of materials at the microscale or nanoscale. Furthermore, the use of light patterns from computer-generated holograms is effective to implement parallel and rapid processing within the irradiation area without scanning. Based on this strategy, fabricating three-dimensional structures is also achievable [10]. These capabilities enable the flexible fabrication and control of materials with complicated shapes.

In this paper, we propose an optical fabrication method for DNA hydrogels. Light irradiation allows for the rapid and flexible patterning of samples. Although photoinduced DNA hydrogels have already been developed [11], control of the shape has not been previously achieved without a mould. Here, we design a scheme to form DNA hydrogels based on the controlled denaturation of double-stranded (ds) DNA via a non-radiative relaxation process of optically excited quenchers [12]. To demonstrate flexible control over the shape, micrometre-sized DNA hydrogels were created by irradiation with specific light patterns.

## 2. Material and methods

**Figure 1.**
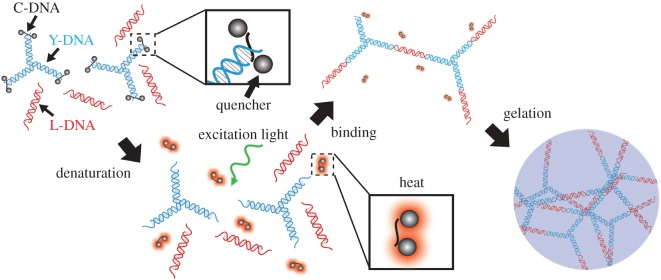
Schematic diagram of the DNA hydrogel formation process.

Figure 1 shows a schematic diagram of the optically induced formation of a DNA hydrogel. The DNA hydrogel consists of Y-motif (Y-DNA) and linker DNA (L-DNA). The Y-DNA and L-DNA are composed of three and two single-stranded DNAs (ssDNAs), respectively (Y-DNA: Y1, Y2, Y3; L-DNA: L1, L2). The sequences of the DNA strands are listed in table 1. Y-DNA segments are connected to each other through the intermediation of L-DNA segments, which is possible because they have complementary pairs of sequences at their sticky ends. For optical control, we introduce a ssDNA called Cap-DNA (C-DNA), which is complementary to the sticky ends of Y-DNAs (8 nt), and modified with quenchers at both ends. After the quenchers become optically excited, thermal energy is released during relaxation. Thus, the irradiated quenchers act as a heat source. This dissipated energy is sufficient to separate an 8 bp dsDNA segment into two ssDNAs [13]. As a result, denaturation of dsDNA via the photothermal effect can be locally induced [14]. Because the quencher is small compared with ssDNA, specific parts of the DNA structure can be controlled. In this paper, Black Hole Quencher 1 (BHQ-1), which has a peak absorption wavelength at 534 nm, is employed as the heat source because of its high quantum yield and short lifetime. In the initial conformation, C-DNAs bind with the sticky ends of the Y-DNAs, which prevent the L-DNAs from linking with the Y-DNAs. After excitation of BHQ-1 by light irradiation, the Y-DNAs dissociate from the C-DNAs and link with the L-DNAs to create a DNA hydrogel. This process is limited to the irradiated region of the excitation light. The yield of the DNA hydrogel depends on the light intensity, so the shape can be flexibly controlled by changing the irradiation pattern.

**Table 1.** DNA sequences and modifications.

strand name	sequence
Y1	5′-CGATTGACCACGCTGTCCTAACCATGACCGTCGAAG-3′
Y2	5′-CGATTGACCTTCGACGGTCATGTACTAGATCAGAGG-3′
Y3	5′-CGATTGACCCTCTGATCTAGTAGTTAGGACAGCGTG-3′
L1	5′-GTCAATCGTCTATTCGCATGAGGATCCCATTCACCGTAAG-3′
L2	5′-GTCAATCGCTTACGGTGAATGGGATCCTCATGCGAATAGA-3′
C-DNA	5′-(BHQ-1)-GTCAATCG-(BHQ-1)-3′

## 3. Results

**Figure 2.**
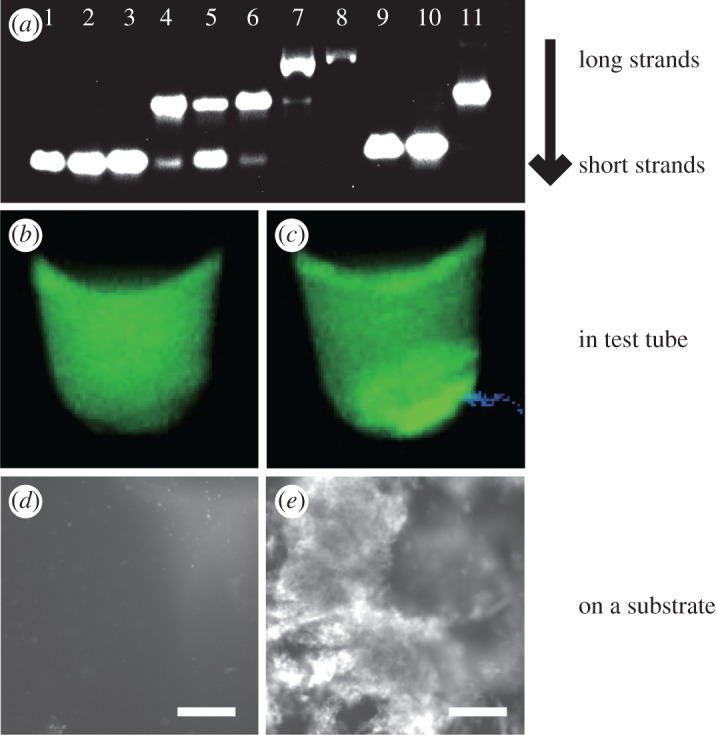
(*a*) Electrophoresis analysis of (1) Y1 (36 nt), (2) Y2 (36 nt), (3) Y3 (36 nt), (4) Y1+Y2 (molar ratio: 1:1), (5) Y2+Y3 (1:1), (6) Y1+Y3 (1:1), (7) Y-DNA (Y1+Y2+Y3, 1:1:1), (8) Y-DNA+C-DNA (1:4), (9) L1 (40 nt), (10) L2 (40 nt), (11) L1+L2 (1:1). Photograph of (*b*) non-irradiated sample and (*c*) irradiated sample under UV irradiation. Fluorescence microscope image of (*d*) non-irradiated sample and (*e*) irradiated sample. Scale bars indicate 50 μm.

First, we investigated the creation of Y-DNA and L-DNA using agarose gel electrophoresis. To test various combinations of Y-DNA, L-DNA and C-DNA, 11 samples were prepared. All DNA strands were purchased from Tsukuba Oligo Service Co. Ltd. The molar concentration of each solution was prepared to be 5.0 μM, and the buffer was composed of H_3_PO_4_ (74 mM) and NaCl (240 mM). The mass concentration of agarose gel was 6.0 wt%, and the electrophoresis was run with a voltage of 120 V for 30 min. The electrophoresis pattern is shown in figure 2*a*. Lanes 1–7 are for Y-DNA, lane 8 is for Y-DNA and C-DNA and lanes 9–11 are for L-DNA. The migration distance in lane 7 is shorter than those in lanes 1–6, which indicates the formation of Y-DNA. Similarly, by comparing the migration distances in lanes 9–11, we confirmed the formation of L-DNA. Moreover, the migration distance in lane 8 is shorter than that in lane 7. The results show that Y-DNA and C-DNA can bind as expected. To investigate the optically induced formation of DNA hydrogels, two test tubes containing the DNA solution with all of the components (6.7 μM Y-DNA, 6.7 μM L-DNA and 26.8 μM C-DNA) in a phosphate buffer (64.8 mM H_3_PO_3_ and 210 mM NaCl) were prepared. One of them was irradiated with light of the excitation wavelength of the quenchers using a diode-pumped solid-state (DPSS) laser (Spectra-Physics KK, Excelsior 532 Single Mode, wavelength: 532 nm, light power: 74.0 mW) for 1 h at 2.3 W cm^−2^ of light intensity. The other test tube was not irradiated. After irradiation, both samples were stained with SYBR green I, which fluoresced (peak emission wavelength = 521 nm) when bound to dsDNA. Because the DNA hydrogels had a high density of aggregated DNA, the fluorescence intensity at locations where the DNA hydrogels existed was expected to increase in the DNA solution. Figure 2*b*,*c* shows the fluorescence images of the non-irradiated and irradiated samples. To observe the fluorescence, the samples were excited using UV irradiation using transilluminator (FUNAKOSHI, NTM-10). Green fluorescence was observed from the entire volume of the non-irradiated sample. By contrast, high fluorescence intensity was observed only from the bottom of the irradiated sample. Each sample was dropped on a slide glass and observed with a fluorescence microscope (objective lens: 10×, camera: Basler, acA1920-25um). Figure 2*d*,*e* shows the recorded images. In the irradiated sample, we found high fluorescence areas (figure 2*e*). By contrast, these regions were not observed in the non-irradiated sample (figure 2*d*). The experimental results show that a DNA hydrogel was created by the light irradiation. Furthermore, such formation was not observed in the DNA solutions without L-DNA or Y-DNA. In our scheme, all the components were required to create the DNA hydrogel.

**Figure 3.**
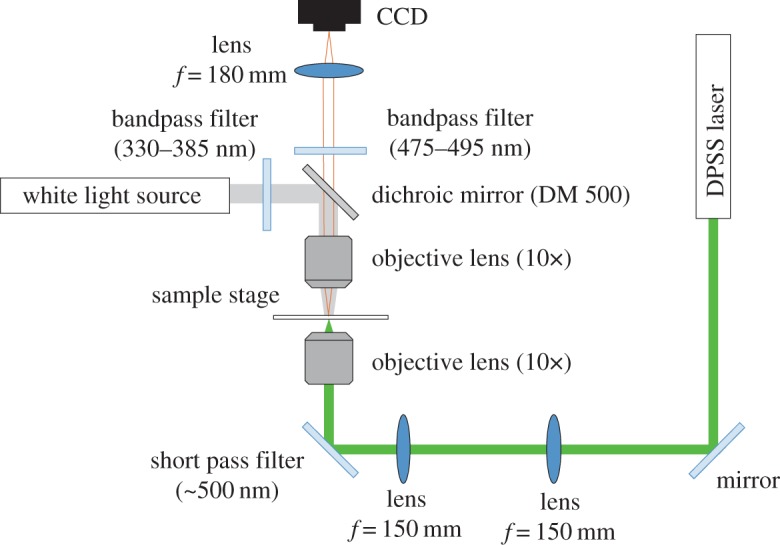
Experimental set-up. *f*, focal length.

**Figure 4.**
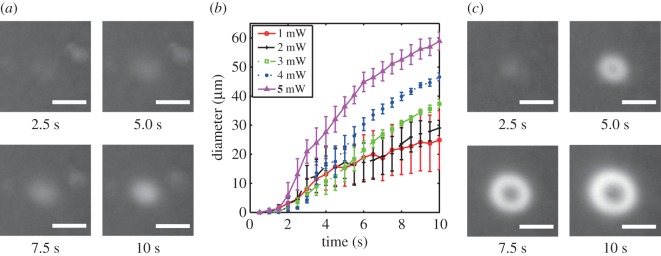
(*a*) Fluorescence images under irradiation with a light spot (3.0 mW, 10.0 s). (*b*) Time dependence of the DNA hydrogel diameters. (*c*) Variation of DNA hydrogel under irradiation. Scale bars indicate 30 μm.

To confirm the spatially controlled gelation by light irradiation and evaluate the localization, we observed the growth of the DNA hydrogels irradiated with a light pattern. In this experiment, the composition of the samples in a buffer (H_3_PO_3_: 3.1 mM, NaCl: 8.5 mM) was 15.0 μM Y-DNA, 13.3 μM L-DNA and 60.0 μM C-DNA. To observe the gelation process, the DNA samples were stained with DAPI (peak excitation wavelength=360 nm, peak emission wavelength=460 nm). DAPI binds to dsDNA segments and emits blue fluorescence. The optical set-up is shown in figure 3. To observe the state of the sample during irradiation, we captured fluorescence images using a charge-coupled device (CCD) camera (Roper Scientific, CoolSNAP fx Monochrome) mounted on a fluorescence microscope (Olympus, BE51WI). To observe the fluorescence of DAPI, a white light source, two bandpass filters (330–385 nm for excitation and 475–495 nm for detection) and a dichroic mirror (DM 500) were employed. Figure 4*a* shows the fluorescence images after irradiation with a focused laser beam (spot diameter=20.8 μm). The fluorescence intensity increased around the centre of the beam. This shows that the DNA hydrogels formed a circular shape, corresponding to the shape of the light spot. However, its area became larger than that of the light spot. This may be due to the thermal or molecular diffusion in the solution of the DNA hydrogels created by the light spot. Figure 4*b* shows the relationship between the diameter of a DNA hydrogel and the irradiation time using 1.0, 2.0, 3.0, 4.0 or 5.0 mW of power. These diameters were measured from the circle-shaped fluorescence area. While the diameters rapidly increased just after initiating irradiation, the rate gradually decreased as the irradiation time became longer. This result shows that the amount of DNA hydrogel created was limited, depending on the intensity. Furthermore, the stronger the light energy was, the more DNA hydrogel was created (see electronic supplementary material, movie S1). The denaturation rate of C-DNA depends on the light intensity, and the amount of DNA hydrogels is limited accordingly. When the irradiation time was long (greater than 4.5 s) and the light power was high (greater than 3.0 mW), the fluorescence intensity decreased at the centre of the irradiation beam, and the DNA hydrogels formed a doughnut-like shape, as shown in figure 4*c* and electronic supplementary material, movie S2. A possible reason is that, due to the strong photoexcitation, BHQ-1 generated enough thermal energy to denature the DNA structure and the DNA hydrogels could not be maintained at the centre of the irradiation area. The high-energy light fabricated unexpected structures of DNA hydrogels, regardless of the intensity distribution. This shows that, in the current set-up, light intensities not exceeding 4.0 mW were suitable for fabricating DNA hydrogels.

**Figure 5.**
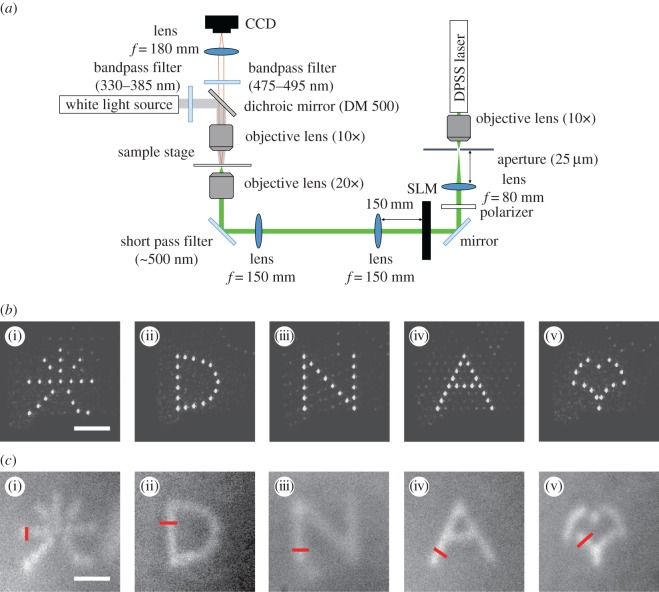
(*a*) Optical set-up. (*b*) light patterns. (i) A Chinese character that means ‘light’, (ii) D, (iii) N, (iv) A, (v) ginkgo leaf shape. (*c*) Fluorescence images after irradiation for 2.5 s. Scale bars indicate 30 μm.

Next, to control the shape of the DNA hydrogels, a light pattern generated using a spatial light modulator (SLM) was used to irradiate a sample. Considering that the formation of DNA hydrogels involves some spread, a spot-array pattern was produced. Figure 5*a* shows the optical set-up for this experiment. By passing the beam through a transmissive liquid crystal SLM (HOLO EYE photonic AG, LC2012, number of pixels: 1024×768, pixel pitch: 36.0 μm), it was modulated in a way that generated a light pattern. The phase distribution was modulated by the SLM, as calculated using the Gerchberg–Saxton algorithm [15]. The beam passed through relay optics, and the Fourier transform pattern was generated at the focal plane of an objective lens to excite the DNA solutions on the stage. The light patterns generated on the sample plane are shown in figure 5*b*. Each light pattern had spots with diameters of approximately 5.0 μm. The average powers over the generated spots were (i) 0.075, (ii) 0.10, (iii) 0.096, (iv) 0.098 and (v) 0.11 mW per spot. The irradiation time was 2.5 s. In this experiment, the concentrations of Y-DNA, L-DNA and C-DNA in a buffer (1.3 mM H_3_PO_3_ and 5 mM NaCl) were 22.2 μM,22.2 μM and 88.8 μM, respectively. Figure 5*c* shows the fluorescence images after irradiation with the individual light patterns. A local increase in the fluorescence intensity corresponding to the irradiated pattern was observed (see electronic supplementary material, movies S3–S7). The shape of a two-dimensional DNA hydrogel can be controlled by light pattern irradiation. The overall size of the DNA hydrogels was 70 μm, which demonstrates the ability of this method to fabricate microscale DNA hydrogels. The widths of these fabricated DNA hydrogels, measured along red lines in figure 5*c*, were (i) 12.1, (ii) 16.1, (iii) 16.1, (iv) 12.2 and (v) 16.2 μm. Differences in the fluorescence intensity were observed in these DNA hydrogels. This may be due to the fact that the light intensity of each spot in the light pattern varies depending on the irradiation position and the number of spots. To reduce variations in the fluorescence intensity, it was necessary to adjust the irradiation time and switch the irradiation patterns. These patterned hydrogels could not be picked up from the sample solution owing to their instability. This is because the concentration of the individual DNA strands was lower than that used in previous research works [1–7,9]. Under such a condition, the DNA density in DNA hydrogels was not enough to maintain the gel structure. Increasing concentration is effective to enhance the stability.

## 4. Conclusion

We presented DNA hydrogels formed by patterned light irradiation. By using the non-radiative relaxation process of quenchers, we demonstrated control of the DNA hydrogel’s shape based on the light pattern. The relationship between the amount of generated DNA hydrogel and irradiation time at each light intensity was determined. Moreover, we found that doughnut-shaped DNA hydrogels were fabricated using a light spot with high energy, regardless of the intensity distribution. Our method enables the formation of structures corresponding to irradiation patterns, and it is expected that the shape of the DNA hydrogels can be flexibly controlled. This should be useful in biomedical applications, such as the fabrication of scaffolds for complicated cell patterns.

## Data accessibility

The relevant data are presented in the electronic supplementary material.
